# Poverty, Cortical Structure, and Psychopathologic Characteristics in Adolescence

**DOI:** 10.1001/jamanetworkopen.2022.44049

**Published:** 2022-11-29

**Authors:** Hannah H. Kim, Katie A. McLaughlin, Lori B. Chibnik, Karestan C. Koenen, Henning Tiemeier

**Affiliations:** 1 Department of Social and Behavioral Sciences, Harvard T. H. Chan School of Public Health, Boston, Massachusetts; 2 Department of Psychology, Harvard University, Cambridge, Massachusetts; 3 Department of Epidemiology, Harvard T. H. Chan School of Public Health, Boston, Massachusetts

## Abstract

**Importance:**

Childhood poverty has been associated with increased internalizing and externalizing problems in adolescence, a period of peak onset for psychiatric problems. The underlying neural mechanisms remain unclear because longitudinal studies of poverty, brain structure, and changes in psychiatric symptoms are lacking.

**Objective:**

To examine whether structural differences in cortical regions mediate the association between household poverty and change in psychiatric symptoms in early adolescence.

**Design, Setting, and Participants:**

This longitudinal cohort study used baseline and 1-year follow-up data from the Adolescent Brain Cognitive Development Study. Children aged 9 to 10 years in the US were enrolled between September 1, 2016, and October 15, 2018. Data analysis was performed from August 13, 2021, to September 30, 2022.

**Exposures:**

Household poverty as measured by income-to-needs ratio, which incorporates family income and adjusts for family size as a percentage of the federal poverty level.

**Main Outcomes and Measures:**

Mediators were children’s cortical surface area, thickness, and volume, obtained using magnetic resonance imaging. Internalizing and externalizing problems at 1-year follow-up were outcomes measured by maternal report using the Child Behavior Checklist. Analyses were adjusted for baseline psychiatric problems and sociodemographic variables, including sex, race and ethnicity, parental educational level, and study site.

**Results:**

Of the 7569 children (mean [SD] age, 9.91 [0.62] years; 3970 boys [52.5%]) included in the analysis, 1042 children (13.8%) lived below the poverty threshold between 2016 and 2018. Poverty was associated with increased externalizing symptoms score at 1-year follow-up (*b* = 1.57; 95% CI, 1.14-1.99), even after adjustment for baseline externalizing symptoms (*b* = 0.35; 95% CI, 0.06-0.64). The longitudinal associations of poverty with increases in externalizing problems over time were mediated by reductions in surface area in multiple cortical regions that support executive functioning (middle frontal gyrus), decision-making (lateral orbitofrontal cortex), visual processing (fusiform gyrus), auditory processing (transverse temporal gyrus), and emotion and language processing (superior temporal gyrus).

**Conclusions and Relevance:**

The findings of this study suggest that childhood poverty is associated with increases in externalizing problems, but not internalizing problems, over time in early adolescence. This association is mediated by reductions in cortical surface area across numerous brain regions. These findings highlight potential neurobiological mechanisms underlying the link between poverty and the emergence of externalizing problems during early adolescence.

## Key Points

**Question:**

Is poverty associated with increased internalizing and externalizing problems in early adolescence, and if so, do cortical morphologic changes mediate the association?

**Findings:**

In a cohort study of 7569 early adolescents aged 9 to 10 years in the Adolescent Brain Cognitive Development Study, living below the federal poverty threshold was associated with an increase in externalizing problems during 1 year. Decreases in cortical surface area and volume of numerous brain regions partially explained this association.

**Meaning:**

The findings of this study suggest a possible pathway from childhood poverty to increased externalizing problems in early adolescents through cortical morphologic changes.

## Teaser

This cohort study uses magnetic resonance imaging of the brain to assess the association of household poverty with psychiatric symptoms in adolescents and to investigate whether the association is mediated by structural differences in cortical regions.

## Introduction

Nearly 10.5 million children in the US lived in poverty and of those, approximately 4.5 million lived in extreme poverty in 2019.^1^ Children raised in poverty are at increased risk for displaying emotional and behavioral problems and psychiatric disorders throughout their development.^2,3^ Such child outcomes are antecedents to mental health problems in adulthood,^4^ along with a host of other adult outcomes including low educational attainment^5^ and unemployment.^6^ Despite numerous studies reporting the association between limited family resources and adolescent outcomes, little is known about the mechanisms underlying the influence of childhood poverty on increased emotional and behavioral problems in early adolescence. In this study, we tested whether cortical structures in several areas of the brain tied to poverty may possibly mediate the association between poverty and psychopathologic symptoms.

Children who are raised in families with lower socioeconomic status (SES) are more likely to develop internalizing symptoms, such as depression and anxiety, and externalizing symptoms, such as aggression and hyperactivity, compared with children raised in more affluent families.^2,3^ Despite these well-documented SES-related differences in emotional and behavioral functioning, the associations between poverty and change in psychiatric symptoms over time are not well understood. There is limited understanding of when in development emotional or behavioral problems are most likely to emerge in children raised in poverty, although many studies have focused specifically on early childhood.^7^ Early adolescence is a window of vulnerability for the emergence of multiple forms of psychiatric problems,^8^ and adolescents living in poverty may be particularly vulnerable. Prior work has observed consistent associations between SES and the presence of mental disorders in adolescents.^2,9,10^ Furthermore, there is evidence that chronic stress, which is associated with poverty, affects structural components of the adolescent amygdala, hippocampal formation, and prefrontal cortex.^11^ As such, poverty may be particularly likely to shape structural brain development during this period, an important time window of neural plasticity.^12,13^

A substantial body of research has investigated the association of SES with brain structure and function in children. In a large study of US children, Noble and colleagues^14^ observed that lower SES was associated with widespread reductions in cortical surface area across numerous brain regions, including frontal, parietal, and temporal association cortex as well as sensorimotor regions. Numerous studies have also documented reductions in hippocampal volume among children with lower SES.^14,15^ The associations of income with brain structure are largest for children in families whose income falls below the poverty line.^14,16,17^ Although growing literature has reported on consistent links between SES and brain structure,^17,18,19^ few studies have looked at the role of brain structure as a possible mechanism linking poverty and increases in psychiatric symptoms during adolescence.

In this study, we used the large, multisite longitudinal Adolescent Brain Cognitive Development (ABCD) Study of early adolescents to investigate associations between poverty, cortical surface area, thickness, and volume.^20^Furthermore, we examined whether associations between poverty and internalizing or externalizing problems were mediated through cortical regions. We corrected for baseline psychiatric symptoms to assess change associated with poverty and brain structure in the early adolescent period. We hypothesized that poverty will be associated with less cortical surface area and smaller cortical volume in widespread regions. We additionally explored whether the brain regions associated with poverty serve as mediators linking poverty to increases in psychiatric symptoms over time.

## Methods

### Study Population

Data were obtained from the ABCD Study, an investigation of child health and neurodevelopment across 21 sites in the US.^20^ Consenting parents and assenting children aged 9 to 10 years were recruited through a school-based probability sampling strategy. We used the ABCD Study Curated Annual Release 3.0, which includes baseline data collected between September 1, 2016, and October 15, 2018, and 1-year follow-up mental health data.^21^ Centralized institutional review board approval was obtained from the University of California, San Diego, and local institutional review boards. This study followed the Strengthening the Reporting of Observational Studies in Epidemiology (STROBE) reporting guideline.

**Figure 1.**
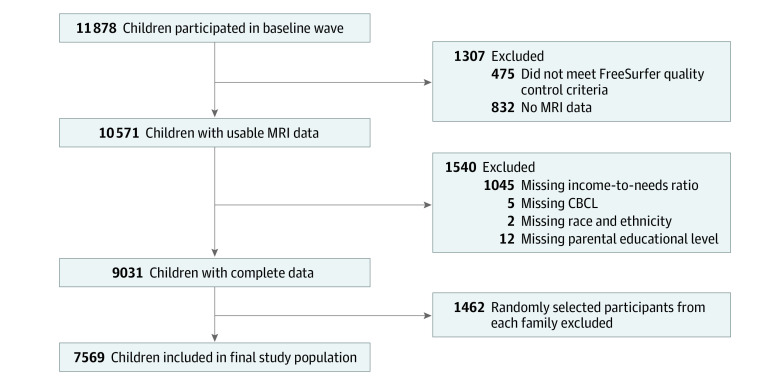
Flowchart of Study Population Selection Individual numbers of participants excluded due to the missingness of the 4 variables do not add up to 1540 because some individuals had missingness in more than 1 of these categories. CBCL indicates Child Behavior Checklist; MRI, magnetic resonance imaging.

Participants were excluded if their brain segmentation did not pass quality control or if exposure, outcome, or covariate data were missing (ie, Child Behavior Checklist score, magnetic resonance imaging assessment, parental educational level, family income, or number of people in the household). One participant was randomly selected per family to account for intrasibling correlations. The final study population consisted of 7569 participants (Figure 1).

### Poverty

Poverty was estimated using baseline income-to-needs ratio (INR), which captures the annual income that a family earns relative to the federal poverty line for a family of that size. Income-to-needs ratio was calculated by dividing total family income by the 2017 poverty threshold provided by the US Department of Health and Human Services.^22^ Total family income and number of persons in the household were caregiver-reported in the parent demographic characteristics survey. Total family income was reported as categorical and was set to the median for each category. A dichotomous measure of poverty reflects financial hardship, while continuous INR reflects resource access across the SES continuum. To capture the impact of financial hardship, a binary poverty variable was created (1 = below, 0 = above threshold). Furthermore, we conducted a secondary analysis using INR as continuous; INR was log-transformed to account for the nonlinear association between INR and psychopathologic characteristics (eFigure 1 in the Supplement).

### Image Acquisition and Processing

Detailed documentation for imaging and preprocessing across the 21 sites have been described previously.^23^ Briefly, each site imaged participants with a 3T scanner using a standard adult-sized coil for the collection of multimodal data: T1- and T2-weighted structural magnetic resonance imaging. Imaging data were processed using FreeSurfer procedures by the ABCD Data Informatics and Resource Center as described in an image processing report.^24^ Quality control procedures were based on automated mean (SD) brain values, and are described by Hagler et al.^24^ We used FreeSurfer, version 6.0 for image processing and estimation of morphometric measures of average cortical measures of surface area, volume, and thickness from the Desikan-Killany-Tourville atlas (eMethods in the Supplement).

### Psychopathologic Symptoms

Emotional and behavioral problems were measured by the parent-reported Child Behavior Checklist for Ages 6-18 at baseline and 1-year follow-up.^25^ Symptoms were rated on a 3-point Likert-type scale. We used raw internalizing and externalizing problem subscale scores.

### Covariates

Child age, sex, child race and ethnicity (Asian, Black, Hispanic, White, and other) as collected in the ABCD study, and highest parental educational level (<high school, high school diploma/general educational development, some college, bachelor’s degree, postgraduate degree) were reported by the primary caregiver. These covariates were considered potential confounders (eFigure 2 and eFigure 3 in the Supplement). We adjusted for race and ethnicity, which are correlated with household income and adolescent psychopathology independently (reflecting structural and societal racism), as a potential confounder. We adjusted for educational level as a confounder because it is likely to be an antecedent of income. Furthermore, to examine whether and how far income in itself makes a difference to adolescents’ outcomes, we adjusted for educational level. We included results without educational level adjustment in eFigure 4 in the Supplement.

### Statistical Analysis

Data analysis was performed from August 13, 2021, to September 30, 2022. Demographic characteristics are described using means (SDs) for continuous variables and frequencies for categorical variables. We investigated whether the association between poverty and psychiatric symptoms was partially accounted for by differences in brain morphologic changes using statistical mediation. First, we examined whether poverty was associated with internalizing and externalizing problems using linear regression, adjusting for the confounders noted above. We then examined potential differences in neural structure by poverty using linear vertex-wise analyses, controlling for the same confounders. Vertex-wise analyses were performed using the *QDECR* R package,^26^ with cortical surface area, thickness, and volume as dependent variables. Correction for multiple testing was applied based on Monte Carlo simulations with a cluster-forming threshold of *P* < .001, which yields false-positive rates that are similar to full permutation testing.^27^ An additional false discovery rate correction (α *P* < .05) was also applied to account for analyzing the left and right hemispheres separately.

Mediational models were run on neural regions that were associated with poverty and internalizing and externalizing symptoms. We tested the indirect outcomes of poverty associated with psychopathologic problems through brain morphologic changes using the *mediation* package in R (R Foundation for Statistical Computing).^28^ This bootstrapping approach with 8000 random samplings provides 95% bias-corrected CIs.^29^ Confidence intervals that do not contain 0 indicate a significant indirect finding. Brain measurements and covariates were collected at baseline. Internalizing and externalizing problems were assessed at 1-year follow-up. The analyses adjusted for exposure-mediator confounders child sex, age, race and ethnicity, parental educational level, and study site. The mediator-outcome confounders included baseline internalizing and externalizing problems in addition to child sex, age, race and ethnicity, parental educational level, and study site. Thus, we examined the association of poverty with psychiatric symptom changes during 1 year and the potential mediating role of brain morphologic changes.

To investigate how much of the total effect operates through the mediators, we ran a single multiple mediator model with structural equation modeling, which allows for the joint estimation of all parameters of a mediation model by allowing the mediators to be conditionally dependent given the exposure and measured covariates without being causally ordered.^30,31^ Through this model, we can estimate the total proportion mediated by all cortical regions. Mediation was performed using a maximum likelihood estimator while bootstrapping 95% bias-corrected CIs. The same set of covariates as the independent mediator models were adjusted for. Goodness of fit was assessed based on root-mean-square error of approximation less than or equal to 0.05, comparative fit index greater than 0.95, and standardized root-mean-square residual less than or equal to 0.08.^32^ All analyses were conducted in R, version 3.6.2. Statistical significance was set at *P* < .05, and all tests were 2-tailed.

## Results

### Sociodemographic Characteristics

Of the 7569 children between ages 9 and 10 years with data on Child Behavior Checklist scores, structural magnetic resonance imaging, and sociodemographic variables, 3599 were girls (47.5%) and 3970 were boys (52.5%); mean (SD) age was 9.91 (0.62) years. A total of 1042 children (13.8%) lived below the poverty line between 2016 and 2018. Table 1 summarizes the analytic population by poverty status.

**Table 1.** Study Population Characteristics for the Overall Sample and by the Federal Poverty Level

Characteristic	No. (%)		
	Participants above poverty threshold (n = 6527)	Participants below poverty threshold (n = 1042)	Overall (n = 7569)
Age, mean (SD)	9.92 (0.62)	9.87 (0.61)	9.91 (0.62)
Sex			
Female	3111 (47.7)	488 (46.8)	3599 (47.5)
Male	3416 (52.3)	554 (53.2)	3970 (52.5)
Race and ethnicity			
Asian	153 (2.3)	6 (0.6)	159 (2.1)
Black	575 (8.8)	376 (36.1)	951 (12.6)
Hispanic	1110 (17.0)	362 (34.7)	1472 (19.4)
White	4035 (61.8)	184 (17.7)	4219 (55.7)
Other^a^	654 (10.0)	114 (10.9)	768 (10.1)
Household income, $			
<50 000	1064 (16.3)	1040 (99.8)	2104 (27.8)
≥50 000-99 900	2195 (33.6)	2 (0.2)	2197 (29.0)
≥100 000	3268 (50.1)	0 (0.0)	3268 (43.2)
Highest educational level			
<HS diploma	68 (1.0)	192 (18.4)	260 (3.4)
HS diploma/GED	296 (4.5)	303 (29.1)	599 (7.9)
Some college	1455 (22.3)	441 (42.3)	1896 (25.0)
Bachelor’s degree	1921 (29.4)	75 (7.2)	1996 (26.4)
Postgraduate degree	2787 (42.7)	31 (3.0)	2818 (37.2)
Symptoms at baseline, mean (SD)			
CBCL internalizing	5.01 (5.37)	5.89 (6.39)	5.13 (5.53)
CBCL externalizing	4.02 (5.26)	6.34 (7.66)	4.34 (5.70)
Symptoms at 12 mo, mean (SD)			
CBCL internalizing	5.20 (5.43)	5.54 (6.29)	5.24 (5.56)
CBCL externalizing	3.82 (5.03)	5.99 (7.50)	4.12 (5.48)

Abbreviations: CBCL, Child Behavior Checklist; GED, general educational development; HS, high school.

^a^ Includes participants of Alaskan Native, American Indian, Native Hawaiian, Pacific Islander, and multiple race.

### Poverty and Psychiatric Symptoms

Poverty was associated with greater externalizing problems at 1 year, after adjusting for covariates (*b* = 1.57; 95% CI, 1.14-1.99; *P* < .001). After additionally adjusting for baseline externalizing problems, we also found poverty to be associated with greater externalizing problems (*b* = 0.35; 95% CI, 0.06-0.64; *P* = .02), suggesting that poverty was associated with greater increases in externalizing problems over time. Poverty was not associated with internalizing symptoms after adjusting for covariates (*b* = 0.41; 95% CI, −0.02 to 0.85; *P* = .06), nor after additionally adjusting for baseline internalizing problems (*b* = −0.14; 95% CI, −0.46 to 0.17; *P* = .40).

### Poverty and Cortical Surface Area

**Figure 2.**
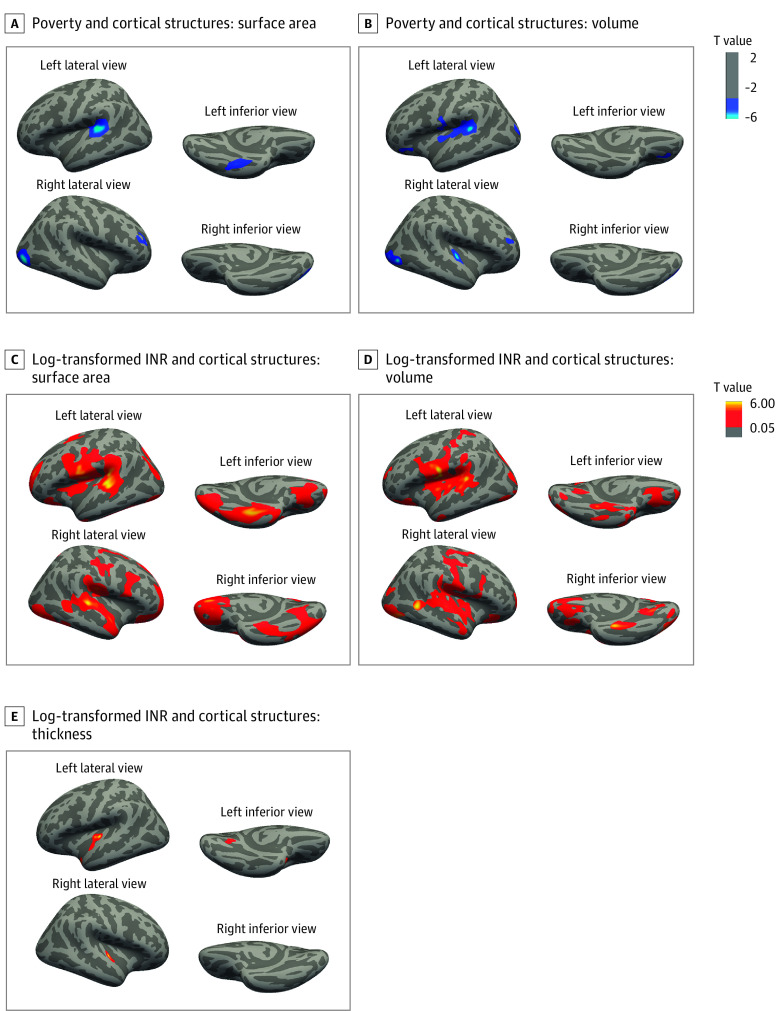
Associations Between Poverty and Income-to-Needs Ratio (INR) and Cortical Morphologic Changes Results from vertex-wise analysis examining the association in the primary analysis between poverty threshold and cortical surface area (A) and volume (B). Cortical thickness is not shown for the primary analysis because there were no significant clusters. Secondary analysis shown for log-transformed INR and cortical surface area (C), volume (D), and thickness (E). The shaded regions in gradient dark blue to light blue denote the magnitude of negative T values that survived multiple testing, with light blue indicating larger T values compared with regions shaded in dark blue. The shaded regions in gradient yellow to red denote the magnitude of positive T values that survived multiple testing, with yellow indicating larger T values compared with regions shaded in red. Models were adjusted for age, race and ethnicity, highest parental educational level, and study site. Statistical details of the clusters are provided in eTable 2 in the Supplement.

Results from the vertex-wise analyses identified 4 regions where cortical surface area was associated with poverty after adjusting for covariates and multiple testing (Figure 2A,B; eTable 1 in the Supplement). Specifically, living in poverty was associated with less cortical surface area in the left superior temporal gyrus (STG), left fusiform gyrus, right lateral occipital cortex, and right middle frontal gyrus (MFG). These regions are involved in auditory and language processing, visual recognition and processing, and executive functioning and decision-making. The associations of poverty with different cortical characteristics were consistent; 3 of the 4 cortical surface regions overlapped with volume.

### Poverty and Cortical Volume

Results from the vertex-wise analyses identified 7 regions in which poverty was associated with smaller cortical volume after adjusting for covariates. Early adolescents exposed to poverty were more likely to have smaller cortical volumes in the left STG, postcentral gyrus, lateral occipital cortex, lateral orbitofrontal cortex (OFC), right lateral occipital cortex, transverse temporal gyrus, and rostral MFG (Figure 2A,B; eTable 1 in the Supplement). After multiple testing correction, no cortical thickness regions were associated with poverty.

### Structural Brain Mediators of the Association of Poverty With Externalizing Symptom Change

Mediation analysis was conducted to determine the extent to which the association between poverty and externalizing behavior was mediated by differences in cortical surface area (Table 2). Indirect associations of poverty with externalizing problems through cortical surface area were observed for left STG, left fusiform gyrus, and right MFG, but not for right lateral occipital cortex. For cortical volume, indirect associations were observed for left STG, left postcentral gyrus, left OFC, right transverse temporal gyrus, and right MFG, but not for left or right lateral occipital cortex.

**Table 2.** Results From Individual Mediation Models Examining the Associations Between Poverty, Cortical Brain Regions, and Externalizing Problems in the ABCD Study^a^^,^^b^

Variable	Indirect effect (95% CI)	Estimated proportion mediated of total effect (%)^c^
Cortical surface area region		
Left hemisphere		
Superior temporal gyrus	0.014 (0.004 to 0.030)	4.1
Fusiform gyrus	0.005 (−0.001 to 0.020)	1.6
Right hemisphere		
Lateral occipital cortex	0.009 (−0.003 to 0.030)	2.5
Rostral middle frontal gyrus	0.009 (0.001 to 0.020)	2.6
Cortical volume region		
Left hemisphere		
Superior temporal gyrus	0.019 (0.007 to 0.040)	5.4
Postcentral gyrus	0.012 (0.002 to 0.030)	3.5
Lateral occipital cortex	0.011 (0.000 to 0.030)	2.5
Lateral orbitofrontal cortex	0.013 (0.002 to 0.030)	3.8
Right hemisphere		
Lateral occipital cortex	0.011 (0.000 to 0.030)	3.3
Transverse temporal gyrus	0.018 (0.006 to 0.040)	5.3
Rostral middle frontal gyrus	0.013 (0.003 to 0.030)	3.7
SEM multiple mediator model^b^^,^^d^		
Total sum of indirect effect	0.008 (0.001 to 0.016)	10.9

Abbreviations: ABCD, Adolescent Brain Cognitive Development; SEM, structural equation modeling.

^a^ Externalizing problem scores were standardized. The bias-corrected 95% CIs were constructed using bootstrap resampling with 8000 iterations. Bias-corrected 95% CIs that do not overlap with 0 indicate an indirect association.

^b^ Adjusted for age, sex, race and ethnicity, highest parental educational level, and study site.

^c^ Proportion mediated was estimated as follows: (natural indirect effect/natural direct effect + natural indirect effect) × 100.

^d^ Figure 3 provides for direct, indirect, and total associations from the SEM multiple mediator model.

**Figure 3.**
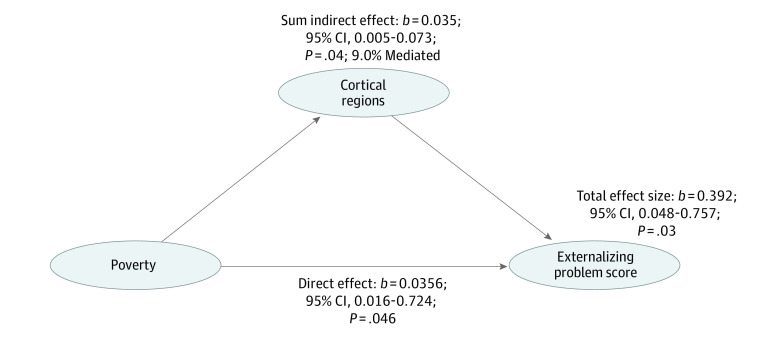
Results From Multiple Mediator Model Using Structural Equation Model A multiple mediator model showed that cortical areas and volume significantly mediated the association between poverty and change in externalizing problem scores by approximately 9%, with sum of indirect association: *b* = 0.035; 95% CI, 0.005-0.073; *P* = .04; direct effect: *b* = 0.356; 95% CI, 0.016-0.724; *P* = .046; and total effect size: *b* = 0.392; 95% CI, 0.048-0.757; *P* = .03.

In the structural equation modeling multiple mediator model that allowed the mediators to be correlated (Figure 3), the total proportion mediated by the cortical surface area and volume regions (ie, total sum of indirect effects) was significant (*b* = 0.392; 95% CI, 0.048-0757; *P* = .03). The sum indirect (*b* = 0.035; 95% CI, 0.005-0.073; *P* = .04) and sum direct associations (*b* = 0.356; 95% CI, 0.016-0.724; *P* = .046) were also significant. The estimated total proportion mediated was 9.0%. Thus, this model suggests that the association of poverty with increased externalizing symptoms over time is partially explained by cortical structure. The model showed good fit according to multiple structural equation modeling fit statistics: root-mean-square error of approximation = 0.021; 95% CI, 0.015-0.027; comparative fit index = 0.99; and standardized root-mean-square residual = 0.010.

### Secondary Analyses

Vertex-wise analyses using income (ie, continuous INR) showed that higher income was associated with larger cortical volume and greater cortical surface area and volume in widespread regions of children’s frontal, temporal, occipital, and parietal lobes (Figure 2C-E). Regions associated with income included the STG, fusiform gyrus, prefrontal cortex, and precentral gyrus–regions linked with various executive functioning and language. In contrast with poverty analyses, which did not result in cortical thickness associations, income was associated with greater cortical thickness in bilateral STG, and in the left lingual gyrus, transverse temporal cortex, and temporal pole.

Although racial and ethnic minority groups had higher rates of poverty (eFigure 5 in the Supplement), associations between poverty and global cortical metrics were invariant across race and ethnicity (eTable 2 in the Supplement).

## Discussion

The current study presents what we believe to be novel results linking poverty, brain structure, and externalizing symptom increases using a large, diverse group of early adolescents. We noted that children experiencing poverty exhibit less cortical surface area and volume compared with their peers across widespread regions in the prefrontal, temporal, and occipital cortex. Furthermore, mediation analyses indicated that the association between poverty and increases in externalizing problems over time was only partially mediated by cortical surface area and volume in specific regions. Taken together, findings suggest that differences in cortical structure are a potential neurodevelopmental mechanism underlying the association of poverty with the emergence of externalizing problems during early adolescence.

Living in poverty was associated with increased externalizing but not internalizing symptoms over time. Consistent with our results, previous studies suggest that poverty is more strongly associated with externalizing than internalizing problems, with effect sizes larger for externalizing than internalizing problems in some studies,^33,34,35,36^ although the magnitude of the difference varies.^3^ Furthermore, our study is consistent with evidence that the association between income and the rate of change in child internalizing symptoms attenuates as children grow older, whereas differences in antisocial behavior between high- and low-income households become more pronounced with time.^37^ A possible explanation is that internalizing problems, such as depression, typically emerge later in adolescence,^8,38^ and because psychiatric symptoms were measured at ages 10 to 11 years, it may be too early to identify differences.

Our findings suggest that poverty is associated with differences in cortical surface area and volume in regions involved in diverse functions, including visual and auditory processing, emotion and language processing, and executive functioning. This pattern of associations in cortical surface area and volume, but not cortical thickness, is consistent with a previous study examining SES and brain structure, which found similar SES associations in widespread regions in surface area and hippocampal volume, but not in cortical thickness.^14^ Increasingly, studies have reported consistent associations between SES and the structure of the lateral prefrontal cortex,^39^ hippocampus,^14,40^ and ventral visual stream.^14^ The regions associated with poverty were also associated with income (modeled continuously), although income was associated with broader and more widespread regions—largely consistent with prior work demonstrating associations between SES and cortical structure across much of the brain.^41^ Our study adds to the notion that the associations of poverty with brain structure are widespread and not localized to any particular region or cortical network.

In our mediation models, we observed that both cortical surface area and volume partially account for the association between poverty and externalizing behaviors in several critical regions of the brain that involve executive functioning (MFG), decision-making (lateral OFC), visual processing (fusiform gyrus), auditory processing (transverse temporal cortex), and emotion and speech processing (STG). These brain regions each explained 2% to 6% of the association between poverty and externalizing problems, and a total of approximately 9% in the multiple mediator model. Due to an expected shared variance among the regions, we expect the total proportion mediated to be smaller than the addition of individual proportions. In the context of population neuroscience studies, this amount of explained variance is not trivial, as externalizing behaviors are undoubtedly multifactorial, with genetic, familial, and social factors each contributing to the emergence of these problems.

Our findings suggest that changes in cortical structure may be one mechanism linking poverty with the emergence of externalizing behaviors in early adolescence. The structural differences associated with poverty could stem from disparities in exposures to environmental toxins, access to enriching cognitive experiences at home and school, neighborhood violence, or differences in stress.^42,43^ These structural differences may then account for behavioral change, since longitudinal studies have demonstrated the link between altered structural brain development and increased externalizing behavior.^44^

### Limitations

This study has limitations. First, poverty and brain structure were measured at the same time. We therefore must assume that poverty is relatively stable and is not a consequence of child behavior. Second, we were unable to examine brain changes over time; future studies should incorporate longitudinal data to understand poverty and trajectories of brain morphologic changes and behavior. Third, we did not examine other forms of adversity, such as abuse or neighborhood-level characteristics that are associated with both poverty and psychiatric symptoms and could contribute to observed associations.^45,46^ Future research will benefit from incorporating other forms of adversity, as well as more proximal environmental factors (eg, child-caregiver relationship). Fourth, it is possible that the observed associations may reflect confounding bias, such as genetic confounding. However, although genome-wide association studies of income have been conducted,^47^ common genetic variants explained only a small proportion of the variance in income. Moreover, income has no clear biological analog and is likely linked to genetic variation via mediated pleiotropy (ie, an interaction of environment, health, and social factors). Fifth, only parent-reported psychopathologic data were available; youth-reported information could yield different results, especially with internalizing problems, which may be less obvious to parents.^48^ Sixth, these results are generalizable to early adolescents; future work should examine whether the mediated outcome is stronger or weaker in earlier or later developmental periods.

## Conclusions

The finding in this study that structural brain differences may mediate the association between poverty and elevated externalizing problems underscores the importance of attention to public health programs policies that target early intervention and prevention. Future work should use longitudinal neuroimaging data to examine the association between poverty, developmental trajectories of cortical structures, and psychopathologic factors.

## Notes

Supplement. **eMethods.** Image Acquisition and Processing **eFigure 1.** Plots of Baseline Income-to-Needs Ratio and 1-Year Follow-up Internalizing and Externalizing Symptoms Showing Nonlinear Associations **eFigure 2.** A Directed Acyclic Graph Depicting Assumptions About the Relationships Between Poverty, Brain Structure, Psychiatric Symptoms, and Potential Confounders **eFigure 3.** Spearman Correlation Matrix Showing the Correlations Between Income to Needs Ratio, Sex, Parental Education, and Internalizing and Externalizing Symptom Scores at Baseline and 1-Year Follow-up **eFigure 4.** Associations Between Poverty and Cortical Morphology Excluding Adjustment for Parental Educational Level **eFigure 5.** Distribution of Income-to-Needs Ratio by Race and Ethnicity **eTable 1.** Associations of Living Below the Federal Household Poverty Threshold With Cortical Morphology Among Preadolescents Aged 9-11 Years **eTable 2.** Results From Linear Regression Models for Total Surface Area, Mean Cortical Thickness, and Total Cortical Volume
