# Supplementary material for: Poverty, Cortical Structure, and Psychopathologic Characteristics in Adolescence
Source: JAMA Netw Open. 2022 Nov 29;5(11):e2244049. doi: 10.1001/jamanetworkopen.2022.44049 (PMC9709650; doi:10.1001/jamanetworkopen.2022.44049)
Supplement: Supplement. — eMethods. Image Acquisition and Processing eFigure 1. Plots of Baseline Income-to-Needs Ratio and 1-Year Follow-up Internalizing and Externalizing Symptoms Showing Nonlinear Associations eFigure 2. A Directed Acyclic Graph Depicting Assumptions About the Relationships Between Poverty, Brain Structure, Psychiatric Symptoms, and Potential Confounders eFigure 3. Spearman Correlation Matrix Showing the Correlations Between Income to Needs Ratio, Sex, Parental Education, and Internalizing and Externalizing Symptom Scores at Baseline and 1-Year Follow-up eFigure 4. Associations Between Poverty and Cortical Morphology Excluding Adjustment for Parental Educational Level eFigure 5. Distribution of Income-to-Needs Ratio by Race and Ethnicity eTable 1. Associations of Living Below the Federal Household Poverty Threshold With Cortical Morphology Among Preadolescents Aged 9-11 Years eTable 2. Results From Linear Regression Models for Total Surface Area, Mean Cortical Thickness, and Total Cortical Volume [file jamanetwopen-e2244049-s001.pdf]

Supplementary Online Content

Kim HH, McLaughlin KA, Chibnik LB, Koenen KC, Tiemeier H. Poverty, cortical structure, and psychopathologic characteristics in adolescence. *JAMA Netw Open*. 2022;5(11):e2244049. doi:10.1001/jamanetworkopen.2022.44049

eMethods. Image Acquisition and Processing

eFigure 1. Plots of Baseline Income-to-Needs Ratio and 1-Year Follow-up Internalizing and Externalizing Symptoms Showing Nonlinear Associations

eFigure 2. A Directed Acyclic Graph Depicting Assumptions About the Relationships Between Poverty, Brain Structure, Psychiatric Symptoms, and Potential Confounders

eFigure 3. Spearman Correlation Matrix Showing the Correlations Between Income to Needs Ratio, Sex, Parental Education, and Internalizing and Externalizing Symptom Scores at Baseline and 1-Year Follow-up

eFigure 4. Associations Between Poverty and Cortical Morphology Excluding Adjustment for Parental Educational Level

eFigure 5. Distribution of Income-to-Needs Ratio by Race and Ethnicity

eTable 1. Associations of Living Below the Federal Household Poverty Threshold With Cortical Morphology Among Preadolescents Aged 9-11 Years

eTable 2. Results From Linear Regression Models for Total Surface Area, Mean Cortical Thickness, and Total Cortical Volume

This supplementary material has been provided by the authors to give readers additional information about their work.

© 2022 Kim HH et al. *JAMA Network Open.*

eMethods. Image Acquisition and Processing

We used FreeSurfer v6.0 for image processing and estimation of morphometric measures of average cortical measures of surface area, volume, and thickness from the Desikan-Killany-Tourville (DKT) atlas. Averages for each cortical parcel was calculated in the default FreeSurfer parcellation scheme using unsmoothed, surface-based maps of morphometric and image intensity measures. For each of the fuzzy-cluster parcels, weighted averages were calculated using smoothed surface maps. Averages of the unsmoothed intensity measures for the volumetric subcortical ROIs and the volume of each structure were calculated. Results were compiled across subjects and summarized in tabulated form.

© 2022 Kim HH et al. *JAMA Network Open.*

eFigure 1. Plots of baseline income-to-needs ratio and one-year follow-up internalizing (A) and externalizing (B) symptoms showing nonlinear associations.

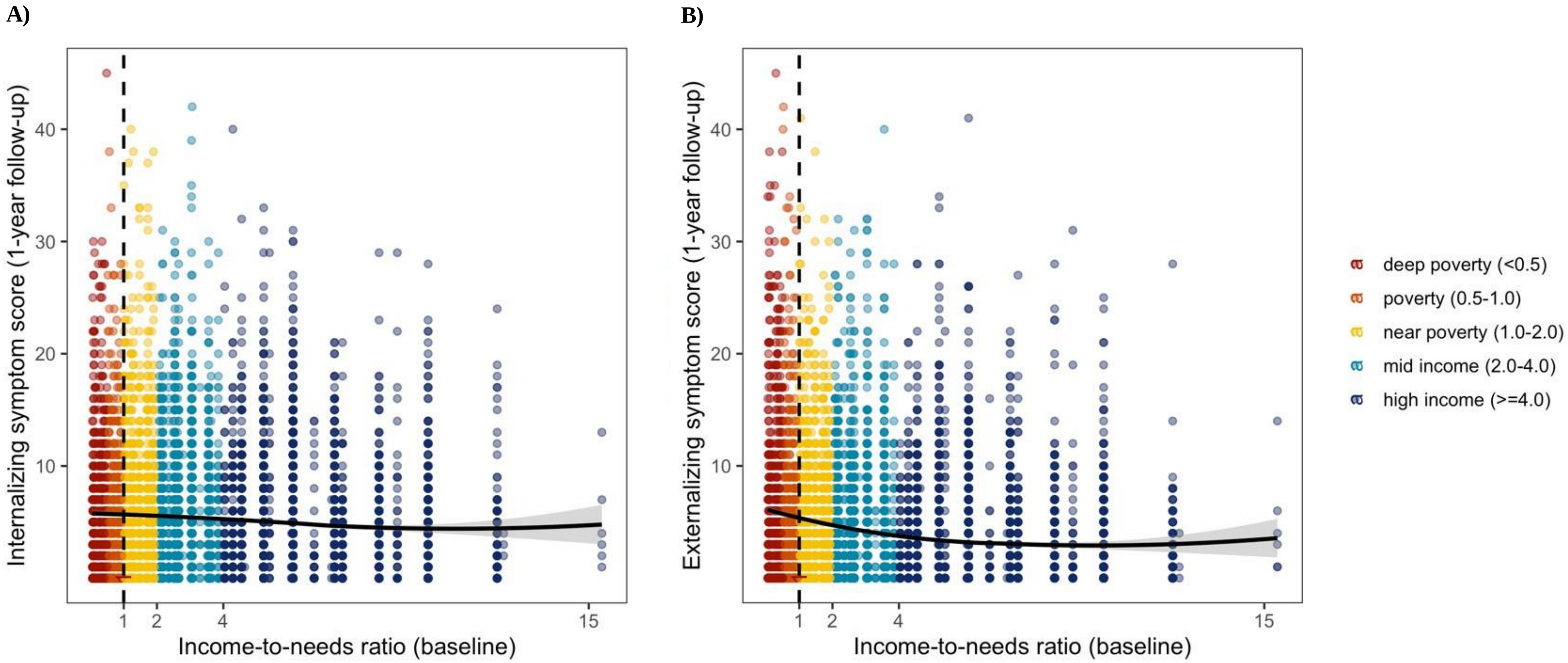

© 2022 Kim HH et al. *JAMA Network Open.*

eFigure 2. A directed acyclic graph depicting assumptions about the relationships between poverty, brain structure, psychiatric symptoms, and potential confounders.

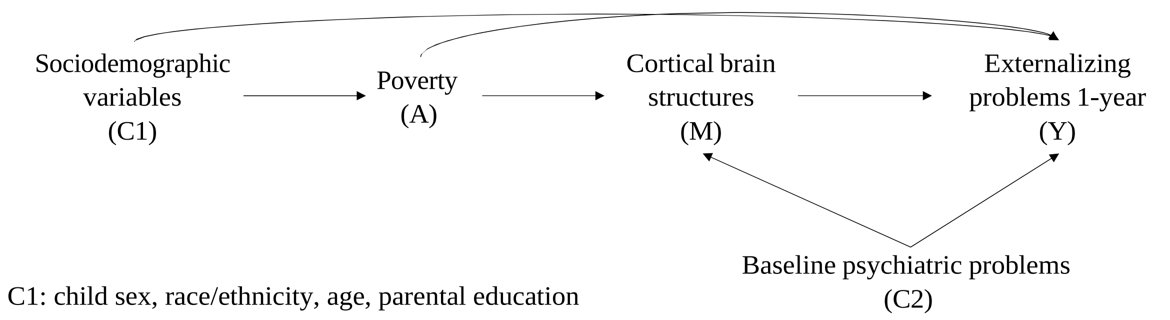

© 2022 Kim HH et al. *JAMA Network Open.*

eFigure 3. Spearman correlation matrix showing the correlations between income to needs ratio, sex, parental education, and internalizing and externalizing symptom scores at baseline and one year follow-up.

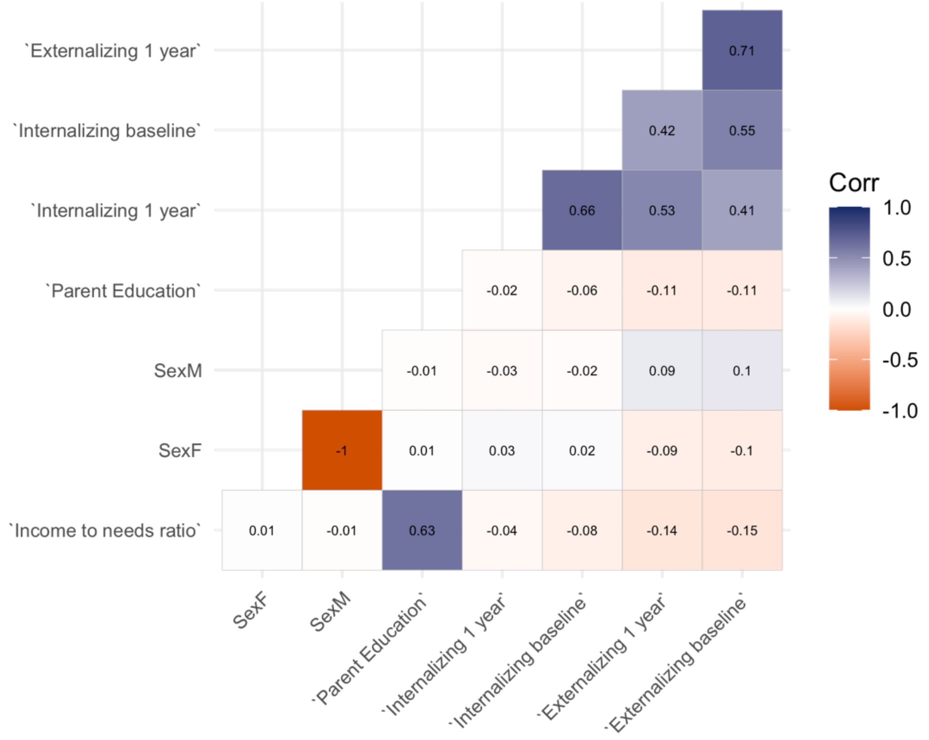

© 2022 Kim HH et al. *JAMA Network Open.*

eFigure 4. Associations between poverty and cortical morphology excluding adjustment for parental educational level. Results from vertex-wise analysis examining the association between poverty and cortical surface area (A), volume (B), and thickness (C). The shaded regions in blue denote the magnitude of ***T* values** from the analyses that survived multiple testing. Models were adjusted for age, race/ethnicity, study site, but not parental education.

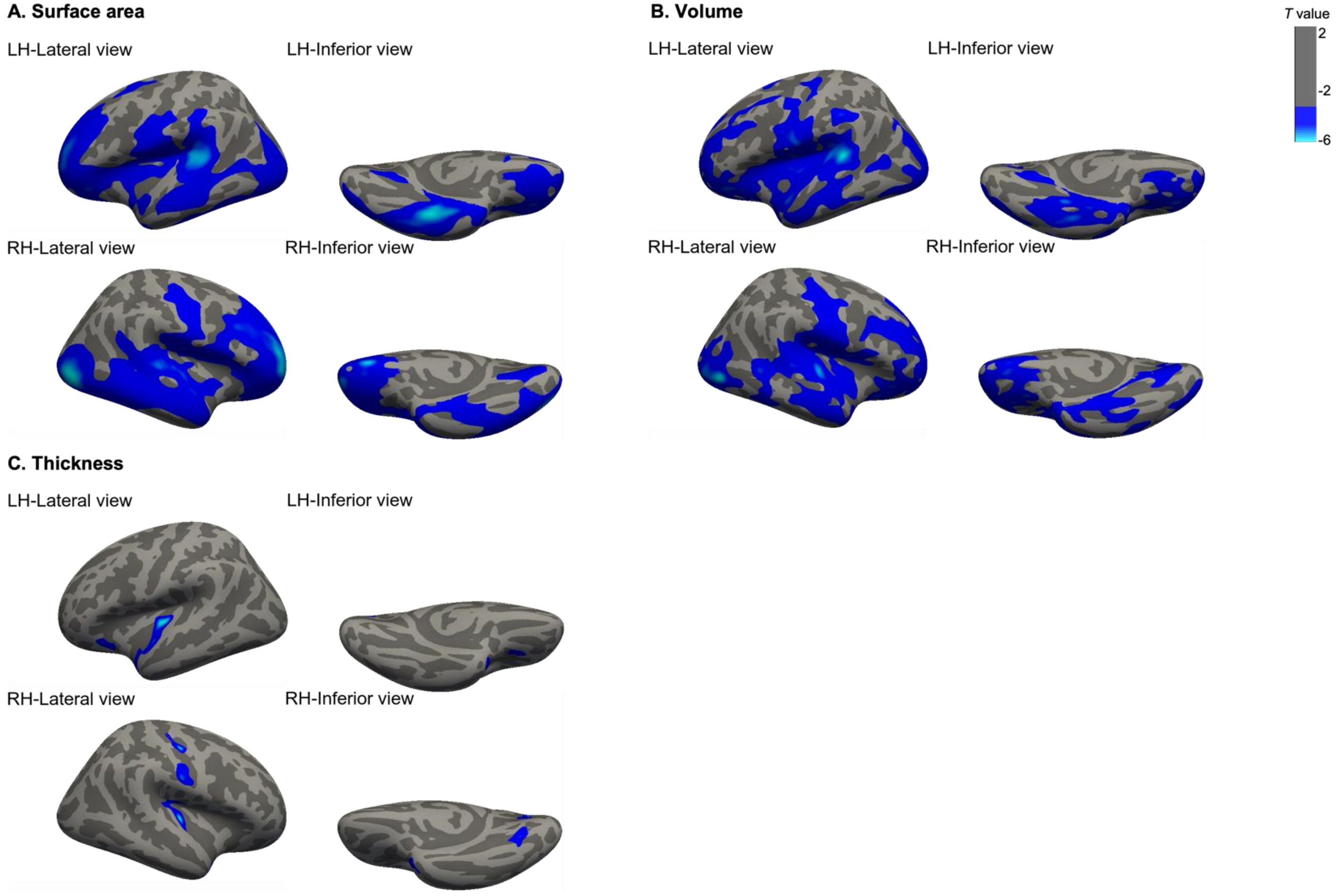

© 2022 Kim HH et al. *JAMA Network Open.*

eFigure 5. Distribution of income-to-needs ratio by race and ethnicity. Other category includes participants of Native Hawaiian, Pacific Islander, Alaskan Native, American Indian, and multiple races.

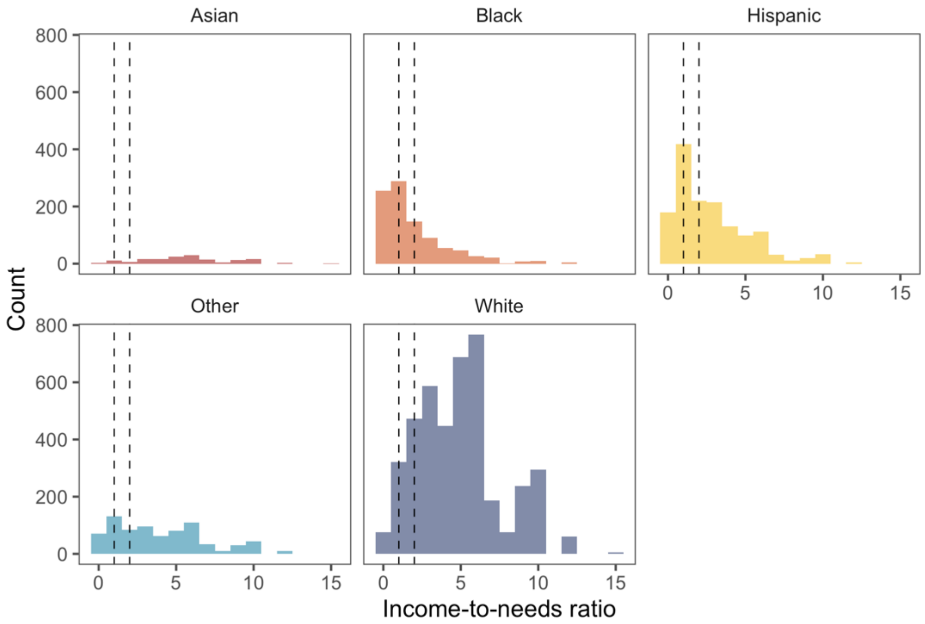

© 2022 Kim HH et al. *JAMA Network Open.*

eTable 1. Associations of living below the Federal household poverty threshold with cortical morphology among pre-adolescents aged 9-11 years

Cortical metrics[a]	Cluster	Hemisphere	Top region 1	Top region 2	Cluster size (mm²)	Mean area (mm²)	Mean volume (mm³)	Mean coef.	Mean SE	Mean t value
**Significant associations from vertex-wise analyses examining poverty and cortical structures that survived multiple testing correction**										
**Surface area**	1	lh	superiortemporal	supramarginal	1183.5	0.641	NA	-0.016	0.004	-4.157
	1	rh	lateraloccipital	NA	1005.2	0.637	NA	-0.024	0.006	-3.805
	2	lh	fusiform	inferiortemporal	525.2	0.659	NA	-0.017	0.005	-3.730
	2	rh	rostralmiddlefrontal	NA	422.2	0.592	NA	-0.024	0.007	-3.376
**Volume**	1	lh	superiortemporal	transversetemporal	1513.4	NA	1.975	-0.043	0.010	-4.050
	1	rh	lateraloccipital	NA	1050.6	NA	1.932	-0.085	0.021	-3.908
	2	lh	postcentral	precentral	358.6	NA	2.092	-0.040	0.011	-3.537
	2	rh	transversetemporal	superiortemporal	356.8	NA	2.153	-0.044	0.011	-3.987
	3	lh	lateraloccipital	inferiorparietal	247.5	NA	2.066	-0.072	0.020	-3.515
	3	rh	rostralmiddlefrontal	NA	222.7	NA	1.755	-0.105	0.029	-3.600
	4	lh	lateralorbitofrontal	parsorbitalis	218.4	NA	1.891	-0.055	0.016	-3.551

Abbreviations: N, number; LH, left hemisphere; RH, right hemisphere, coef., regression coefficient, N vertices, number of vertices in cluster, SE, standard error; NA, not applicable.

[a]Vertex-wise linear regression models adjusted for child sex, race/ethnicity, highest parental education, and study site. All clusters shown survived a cluster-wise Monte Carlo simulation with 5000 iterations correction for multiple comparisons. Cortical thickness not shown because there were no significant clusters. The corresponding brain regions are shown in Figure 2.

© 2022 Kim HH et al. *JAMA Network Open.*

eTable 2. Results from linear regression models for total surface area, mean cortical thickness, and total cortical volume. We observe that none of the interaction terms between poverty and race/ethnicity are significant (except for poverty*other category for total surface area and mean cortical thickness). Note that all three models also adjusted for sex, parental education, and study site (not shown in table for improved readability). Note that 'other' category includes participants of Native Hawaiian, Pacific Islander, Alaskan Native, American Indian, and multiple races.

**Total surface area (mm^2)**			
**Variable**	**Estimate**	**95% CI**	**p-value**
Poverty	-2002	-4235, 232	0.079
Race/ethnicity			
White	—	—	
Asian	-6020	-8454, -3586	<0.001
Black	-12292	-13652, -10932	<0.001
Hispanic	-4576	-5701, -3451	<0.001
Other	-3380	-4614, -2145	<0.001
Poverty * Race/ethnicity			
Poverty * Asian	9657	-2532, 21846	0.12
Poverty * Black	1192	-1732, 4116	0.4
Poverty * Hispanic	590	-2249, 3429	0.7
Poverty * Other	-3746	-7394, -98	0.044
**Mean cortical thickness (mm)**			
**Variable**	**Estimate**	**95% CI**	**p-value**
Poverty	-0.02	-0.03, -0.01	0.004
Race/ethnicity			
White	—	—	
Asian	-0.04	-0.06, -0.03	<0.001
Black	-0.05	-0.05, -0.04	<0.001
Hispanic	-0.03	-0.03, -0.02	<0.001
Other	-0.02	-0.03, -0.02	<0.001
Poverty * Race/ethnicity			
Poverty * Asian	0.02	-0.06, 0.09	0.7
Poverty * Black	0.01	-0.01, 0.03	0.3
Poverty * Hispanic	0.01	-0.01, 0.03	0.2
Poverty * Other	0.03	0.00, 0.05	0.029

© 2022 Kim HH et al. *JAMA Network Open.*

Total cortical volume (mm^3)			
**Characteristic**	**Beta**	**95% CI**	**p-value**
Poverty	-10870	-18020, -3720	0.003
Race/ethnicity			
White	—	—	
Asian	-30540	-38331, -22750	<0.001
Black	-48501	-52855, -44148	<0.001
Hispanic	-21243	-24844, -17641	<0.001
Other	-16245	-20197, -12293	<0.001
Poverty * Race/ethnicity<br>Poverty * Asian	30336	-8685, 69357	0.13
Poverty * Black	5974	-3388, 15335	0.2
Poverty * Hispanic	4875	-4213, 13963	0.3
Poverty * Other	-5872	-17550, 5807	0.3

© 2022 Kim HH et al. *JAMA Network Open.*
